# Supplementary figures and images for: Cullin-RING ligase BioE3 reveals molecular-glue-induced neosubstrates and rewiring of the endogenous Cereblon ubiquitome
Source: Cell Commun Signal. 2025 Feb 19;23:101. doi: 10.1186/s12964-025-02091-5 (PMC11841277; doi:10.1186/s12964-025-02091-5)

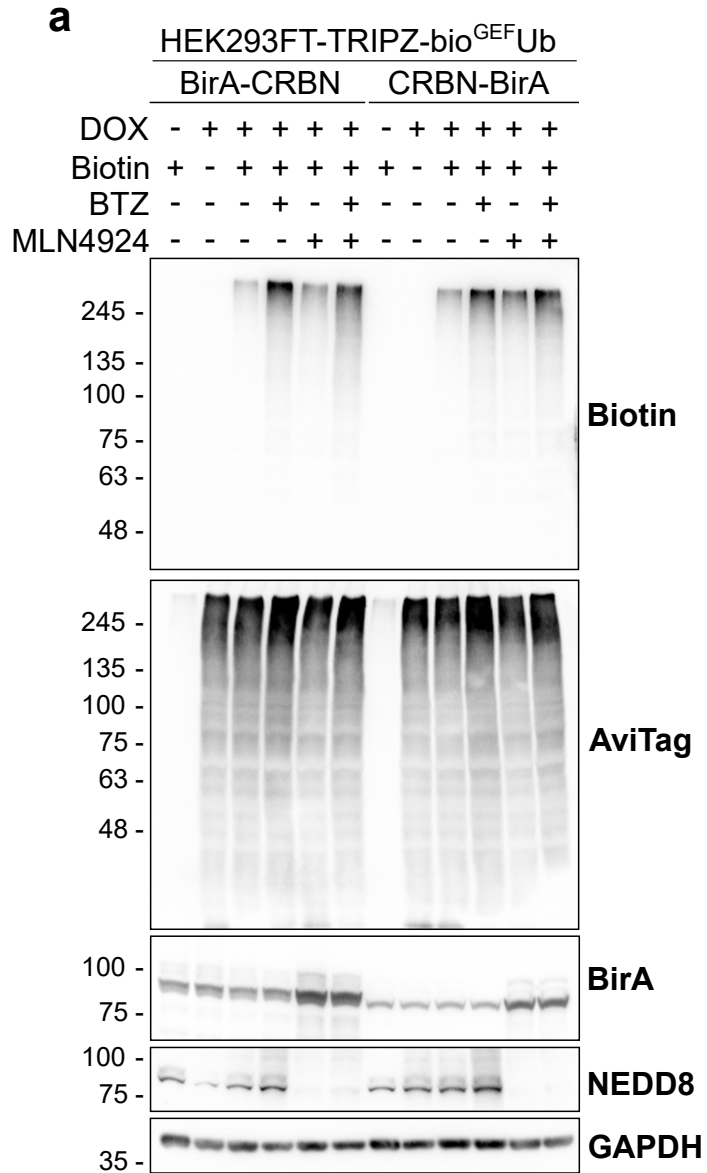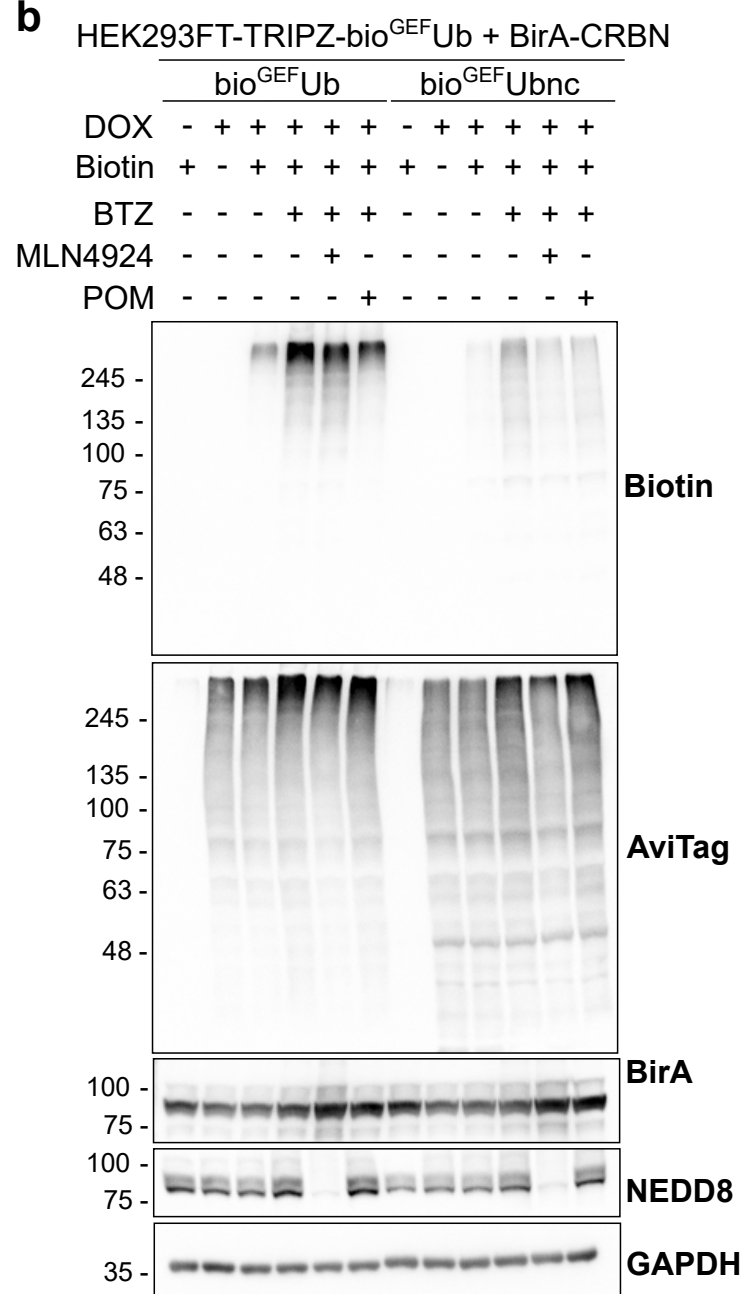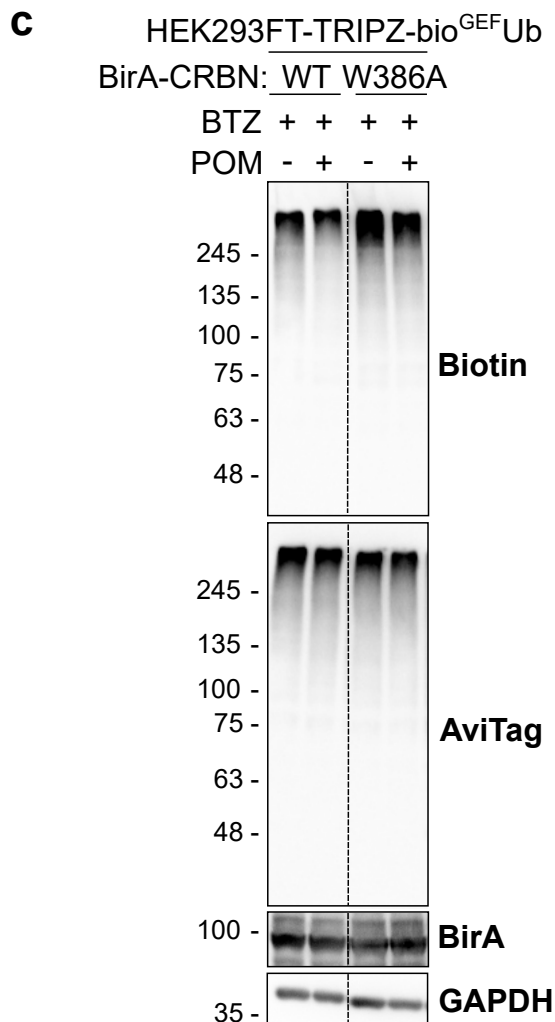

Supplement: Supplementary file 1 — Supplementary Material 1. Fig. S1. Optimization of the experimental conditions for CRBN BioE3. a, b, c) Western blot of BioE3 experiments performed on HEK293FT stable cell lines expressing TRIPZ-bioGEFUb or TRIPZ-bioGEFUbnc and transfected with EFS-BirA-CRBN, EFS-CRBN-BirA or EFS-BirA-CRBNW386A. Indicated samples were induced with doxycycline at 1 µg/ml for 24 hours, treated with 200 nM bortezomib for 6 hours, 1 µM MLN4924 for 24 hours or 10 µM pomalidomide for 6 hours and supplemented with 50 µM biotin for 2 hours. Molecular weight markers are shown to the left of the blots in kDa, antibodies used are indicated to the right. All BioE3 experiments were performed by pre-incubating the cells in dialyzed FBS-containing media prior to transfections. [file 12964_2025_2091_MOESM1_ESM.pdf]

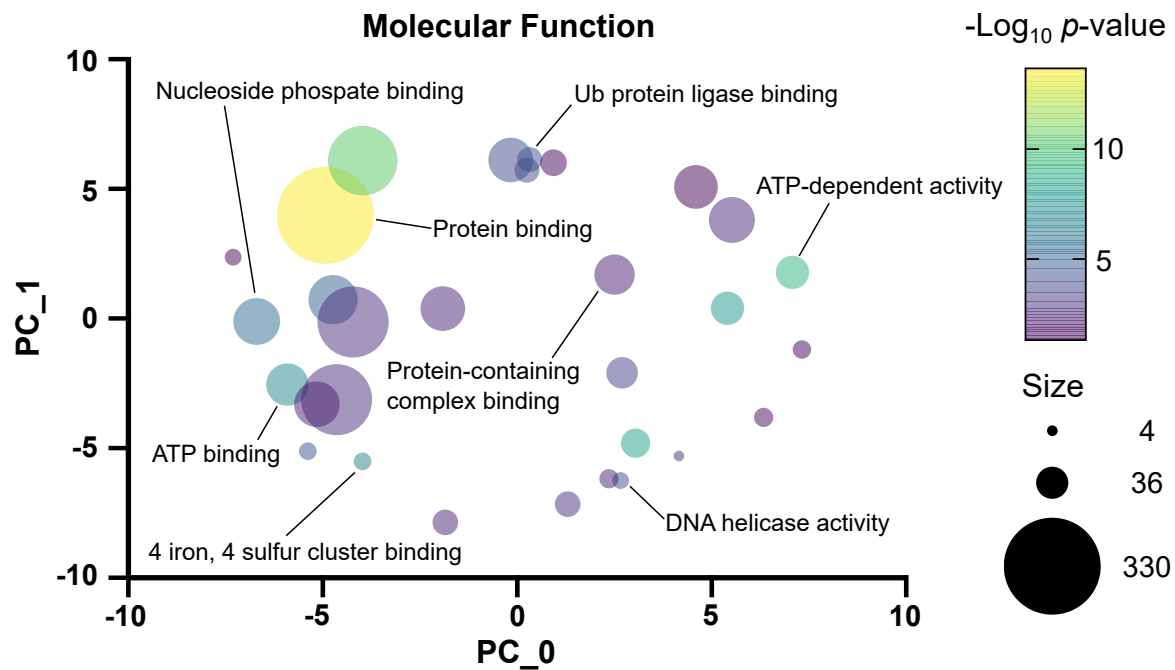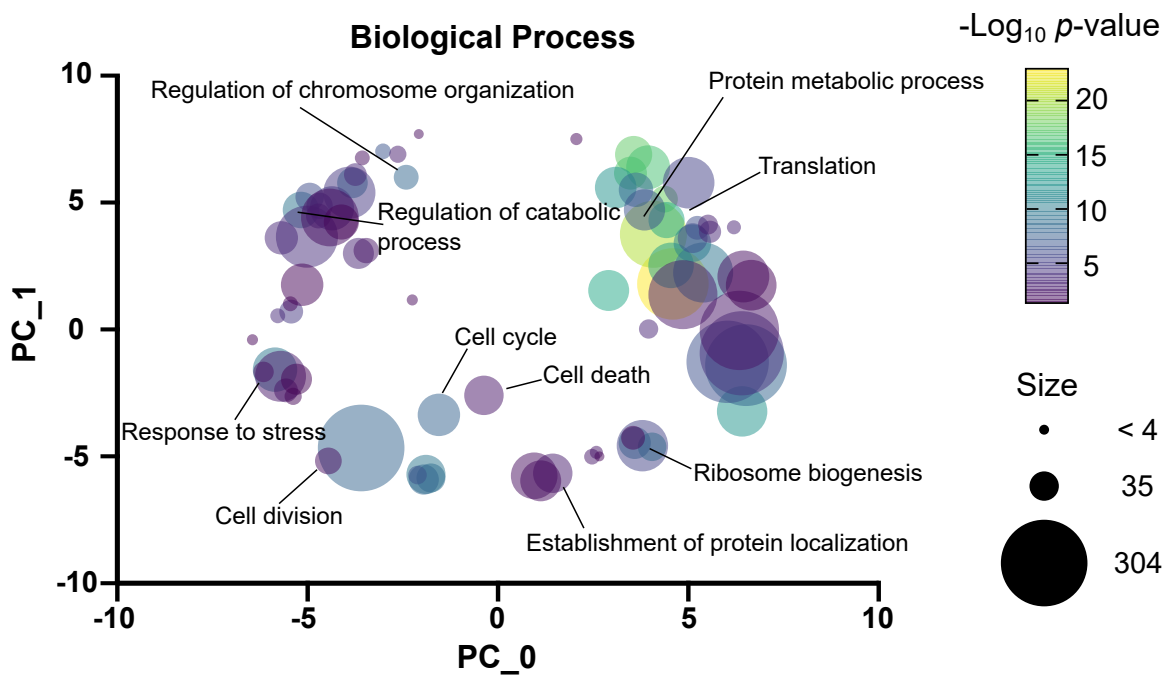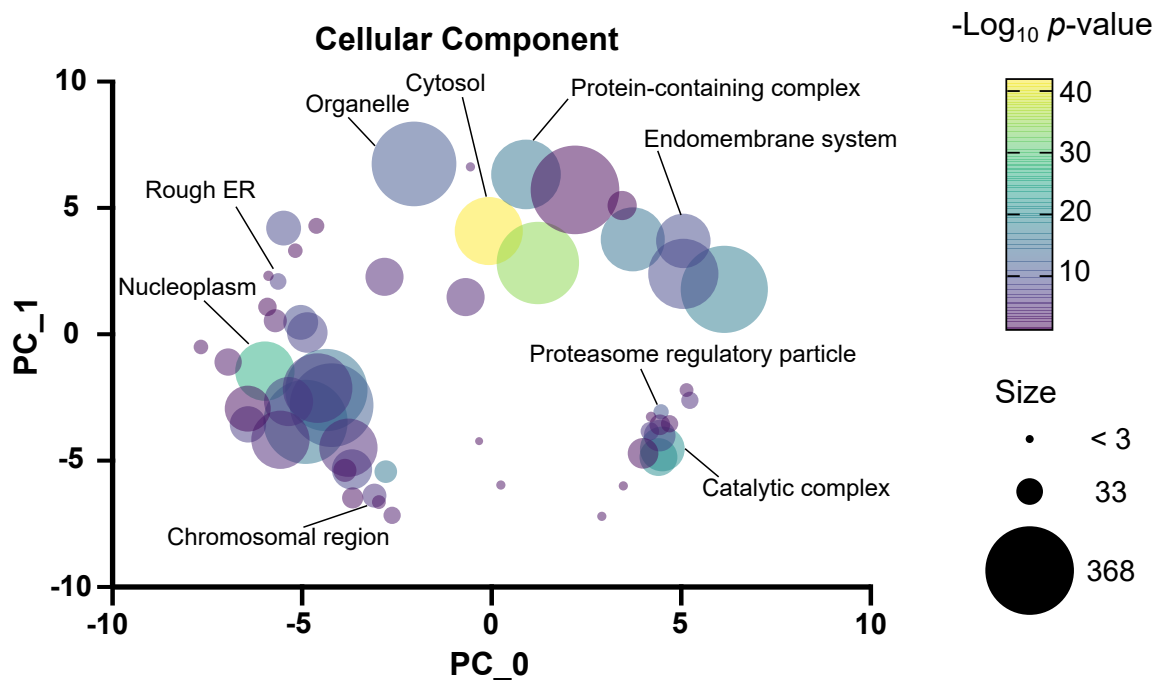

CRBN BioE3 BTZ/ DMSO

Supplement: Supplementary file 3 — Supplementary Material 3. Fig. S3. Gene ontology scatterplot of the CRBN ubiquitinated substrates. REVIGO plots were generated for targets defined in Figure 3b. Colors indicate the -Log10 p-value as shown in the Figure, and size of the bubble indicates the size of each term. Only terms with a p-value < 0.05 are represented. [file 12964_2025_2091_MOESM3_ESM.pdf]

STRING network core cluster (64%)

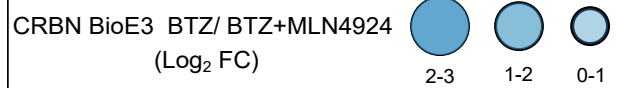

Supplement: Supplementary file 4 — Supplementary Material 4. Fig. S4. STRING network analysis of CRBN NEDDylation-dependent substrates. Substrates defined in Figure 3c show a highly interconnected network composed of 64% of the proteins. Highly interconnected sub-clusters were derived and characterized using MCODE. Color, transparency and size of the nodes were discretely mapped to the Log2 enrichment value as indicated. [file 12964_2025_2091_MOESM4_ESM.pdf]

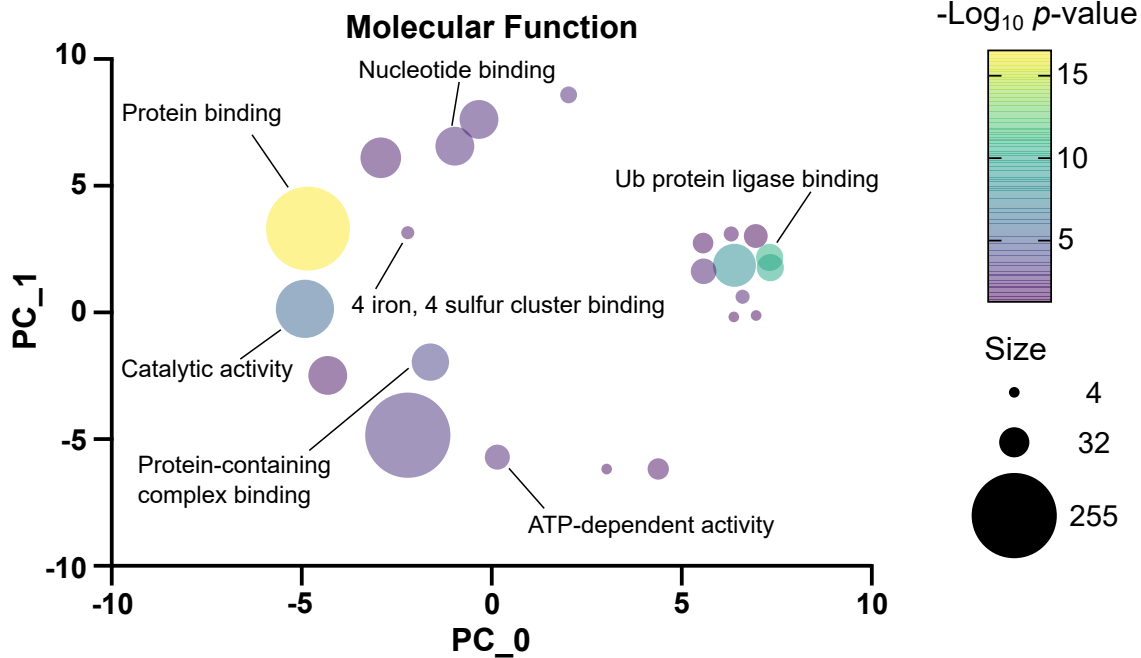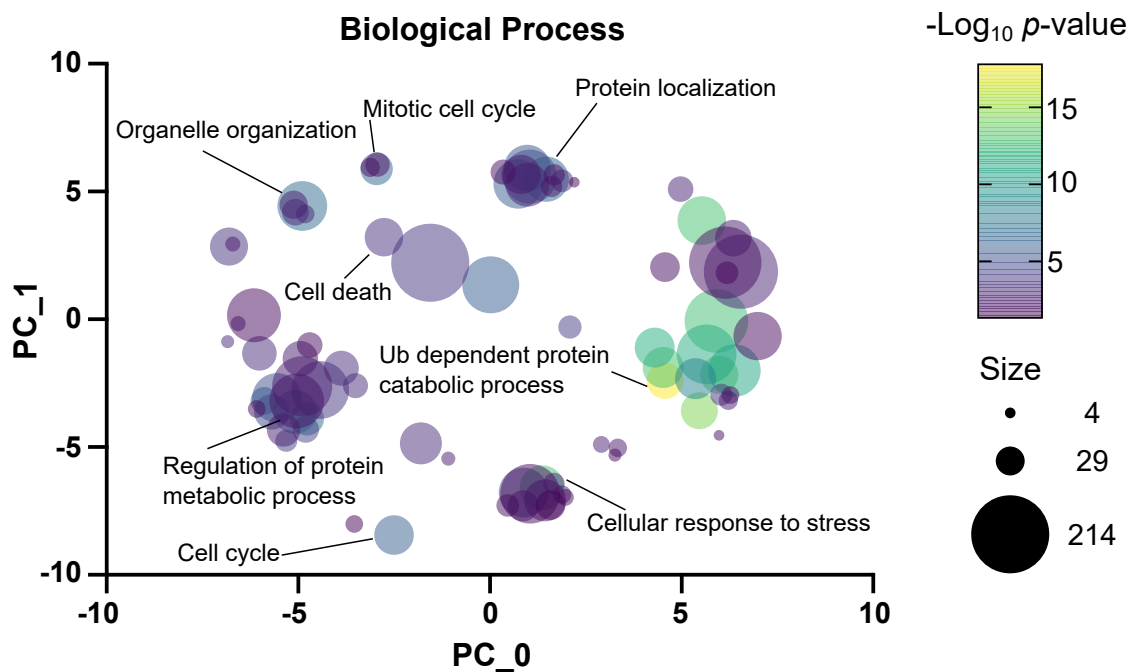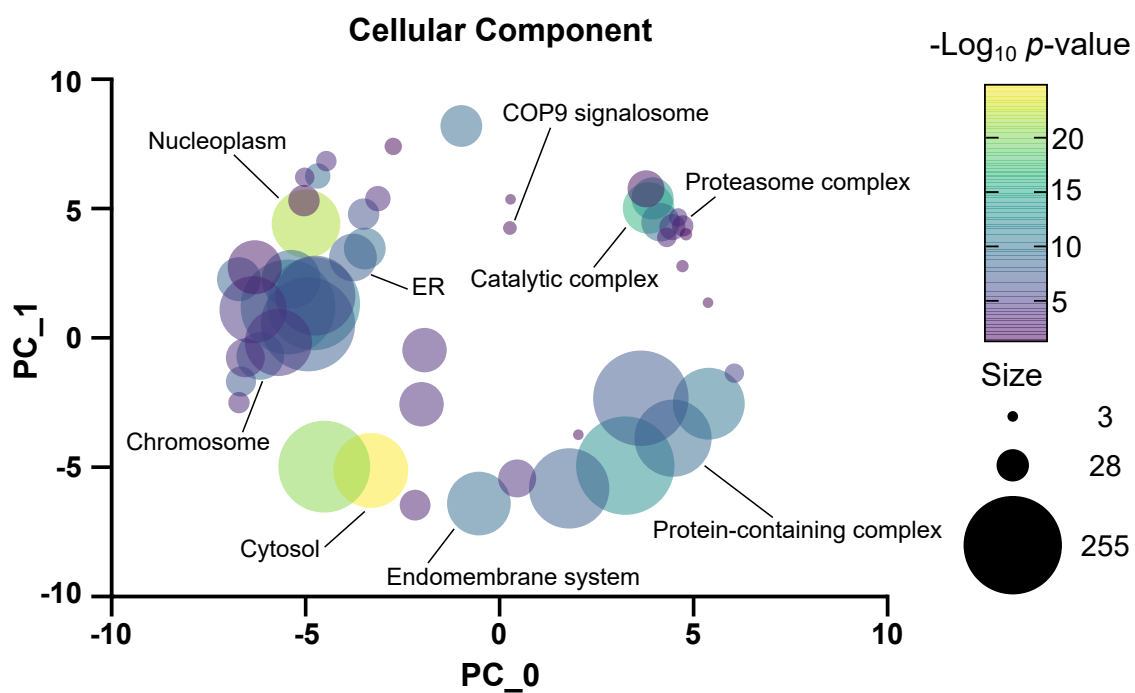

CRBN BioE3 BTZ/ BTZ+MLN4924

Supplement: Supplementary file 5 — Supplementary Material 5. Fig. S5. Gene ontology scatterplot of CRBN NEDDylation-dependent substrates. REVIGO plots were generated for targets defined in Figure 3c. Colors indicate the -Log10 p-value as shown in the Figure, and size of the bubble indicates the size of each term. Only terms with a p-value < 0.05 are represented. [file 12964_2025_2091_MOESM5_ESM.pdf]

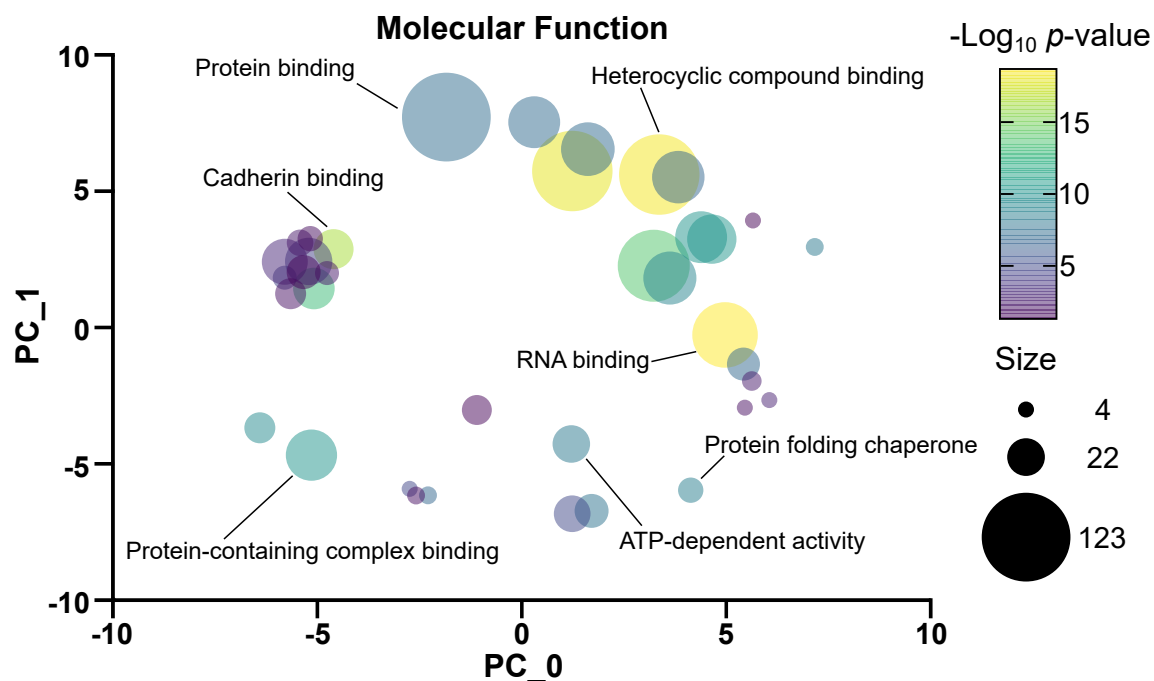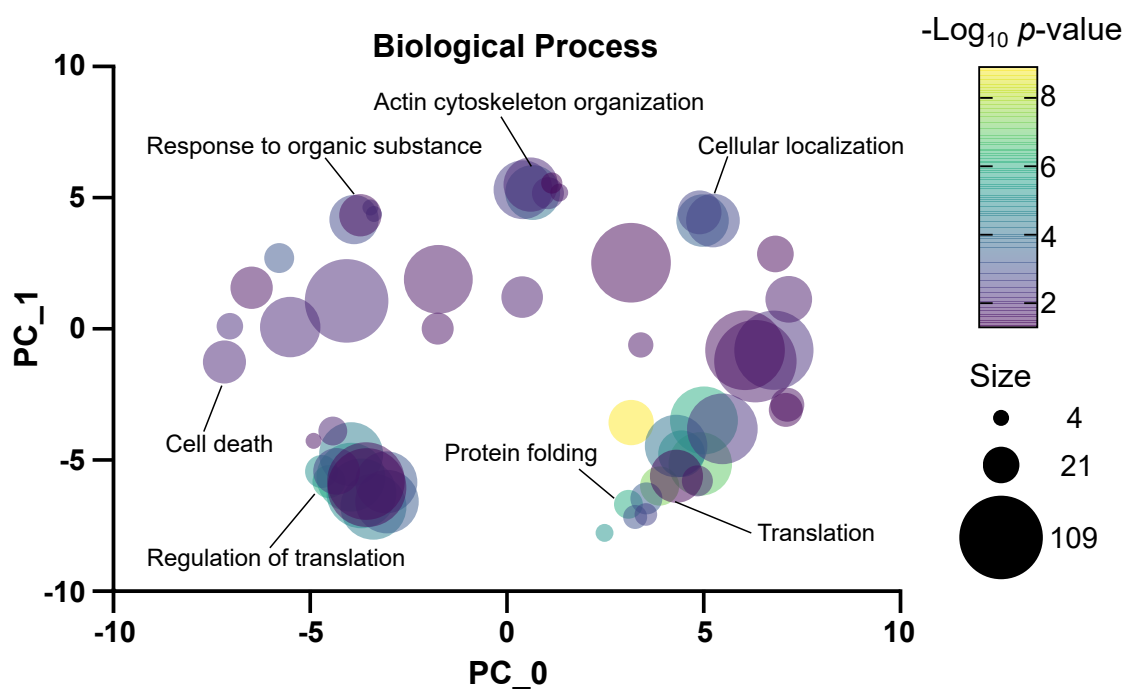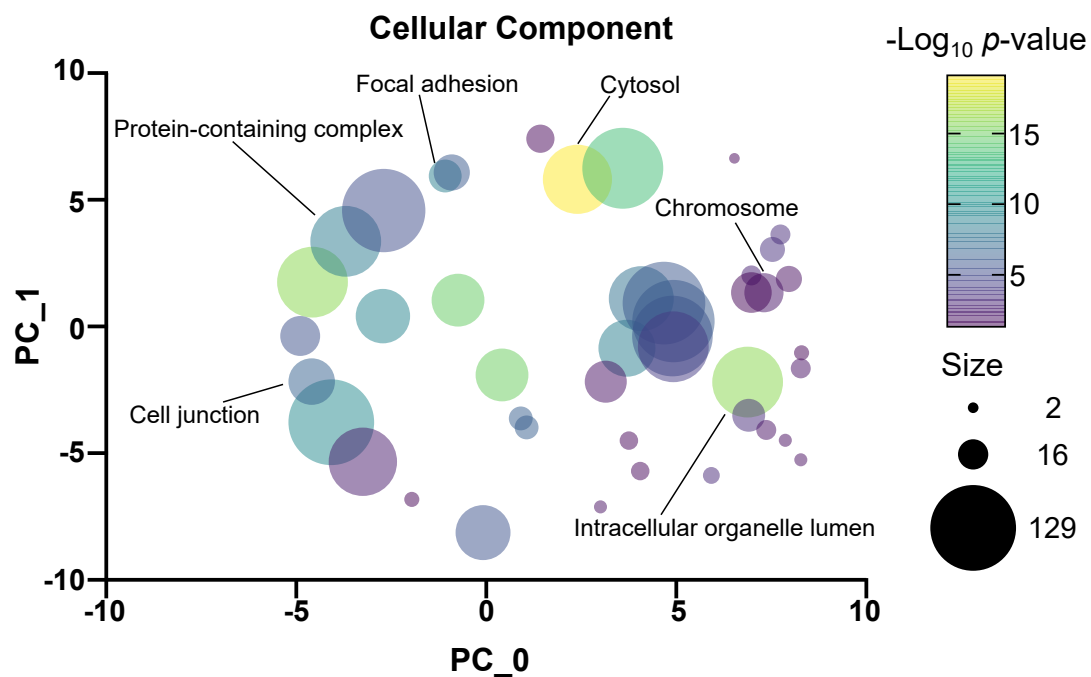

CRBN BioE3 POM+BTZ/ BTZ

Supplement: Supplementary file 7 — Supplementary Material 7. Fig. S7. Gene ontology scatterplot of the neosubstrates of CRBN. REVIGO plots were generated for targets defined in Figure 4a. Colors indicate the -Log10 p-value as shown in the Figure, and size of the bubble indicates the size of each term. Only terms with a p-value < 0.05 are represented. [file 12964_2025_2091_MOESM7_ESM.pdf]

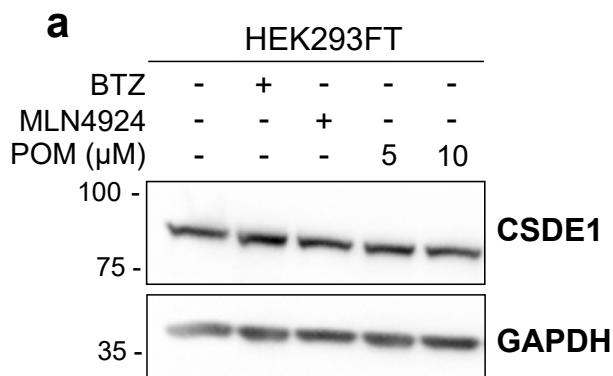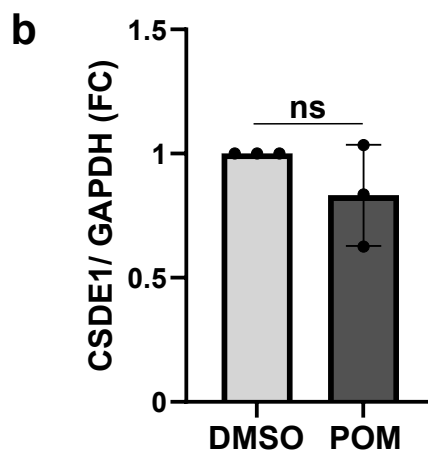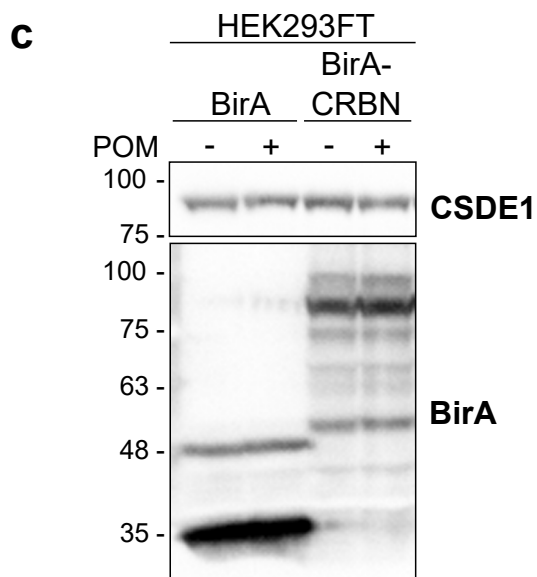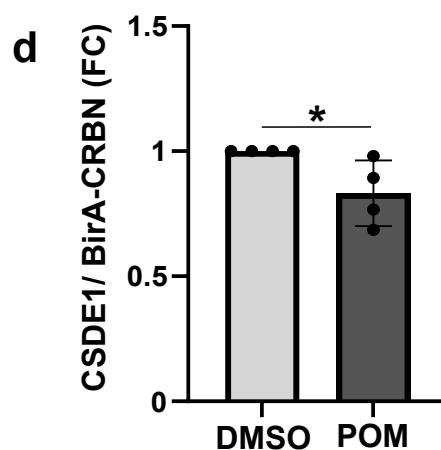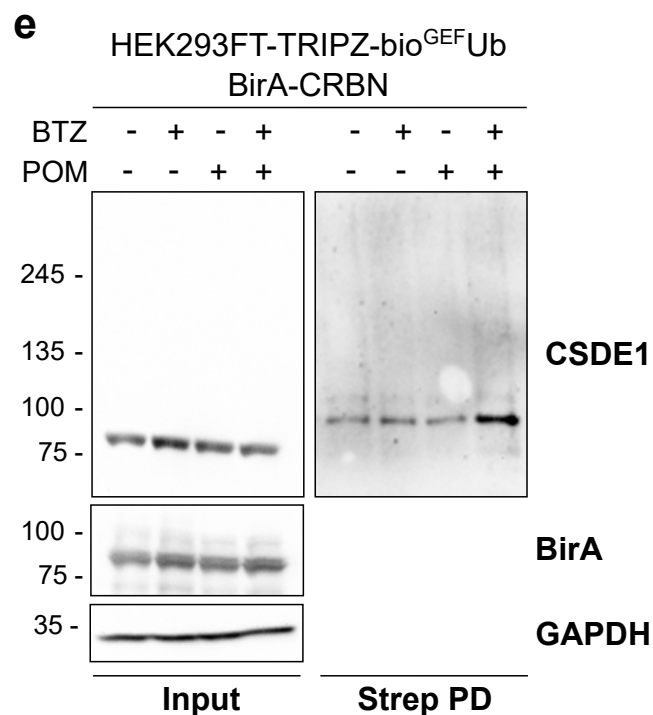

Supplement: Supplementary file 8 — Supplementary Material 8. Fig. S8. Effect of pomalidomide on endogenous protein levels of CSDE1. a) Endogenous CSDE1 levels in HEK293FT cells upon bortezomib, MLN4924 and pomalidomide treatments. b) Quantitative data and statistical analysis of three independent experiments as in Figure S8a. CSDE1 levels in cells treated with DMSO or pomalidomide 10 µM were taken in consideration for the analysis. c) Endogenous CSDE1 levels in HEK293FT cells transiently transfected with EFS -BirA or EFS-BirA-CRBN. d) Quantitative data and statistical analysis of four independent experiments overexpressing BirA-CRBN as in Figure S8c. CSDE1 levels were normalized to BirA-CRBN expression. e) BioE3 experiment performed using HEK293FT stable cells that express TRIPZ-bioGEFUb transiently transfected with BirA-CRBN. BTZ and POM treatments were performed as indicated. Biotinylated proteins were purified using streptavidin beads. Molecular weight markers are shown to the left of the blots in kDa, antibodies used are indicated to the right [file 12964_2025_2091_MOESM8_ESM.pdf]
