# Supplementary material for: Cullin-RING ligase BioE3 reveals molecular-glue-induced neosubstrates and rewiring of the endogenous Cereblon ubiquitome
Source: Cell Commun Signal. 2025 Feb 19;23:101. doi: 10.1186/s12964-025-02091-5 (PMC11841277; doi:10.1186/s12964-025-02091-5)

Fig. 1c

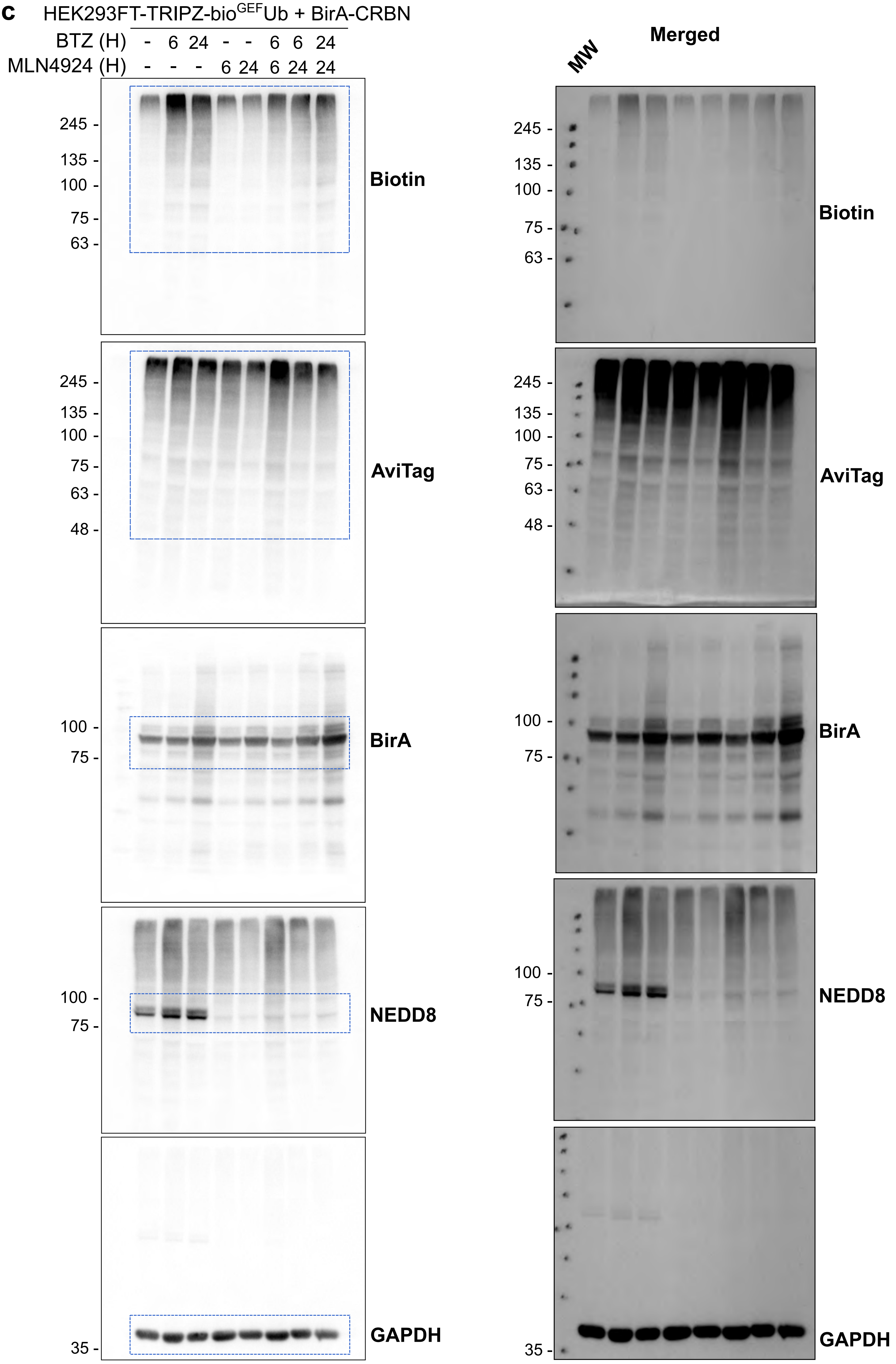

HEK293FT-TRIPZ-bio^{GEF}Ub + BirA-CRBN
BTZ (H) - 6 24 - - 6 6 24
MLN4924 (H) - - - 6 24 6 24 24
Merged
MW
245
135
100
75
63
48
35
Biotin
AviTag
BirA
NEDD8
GAPDH

Fig. 2b

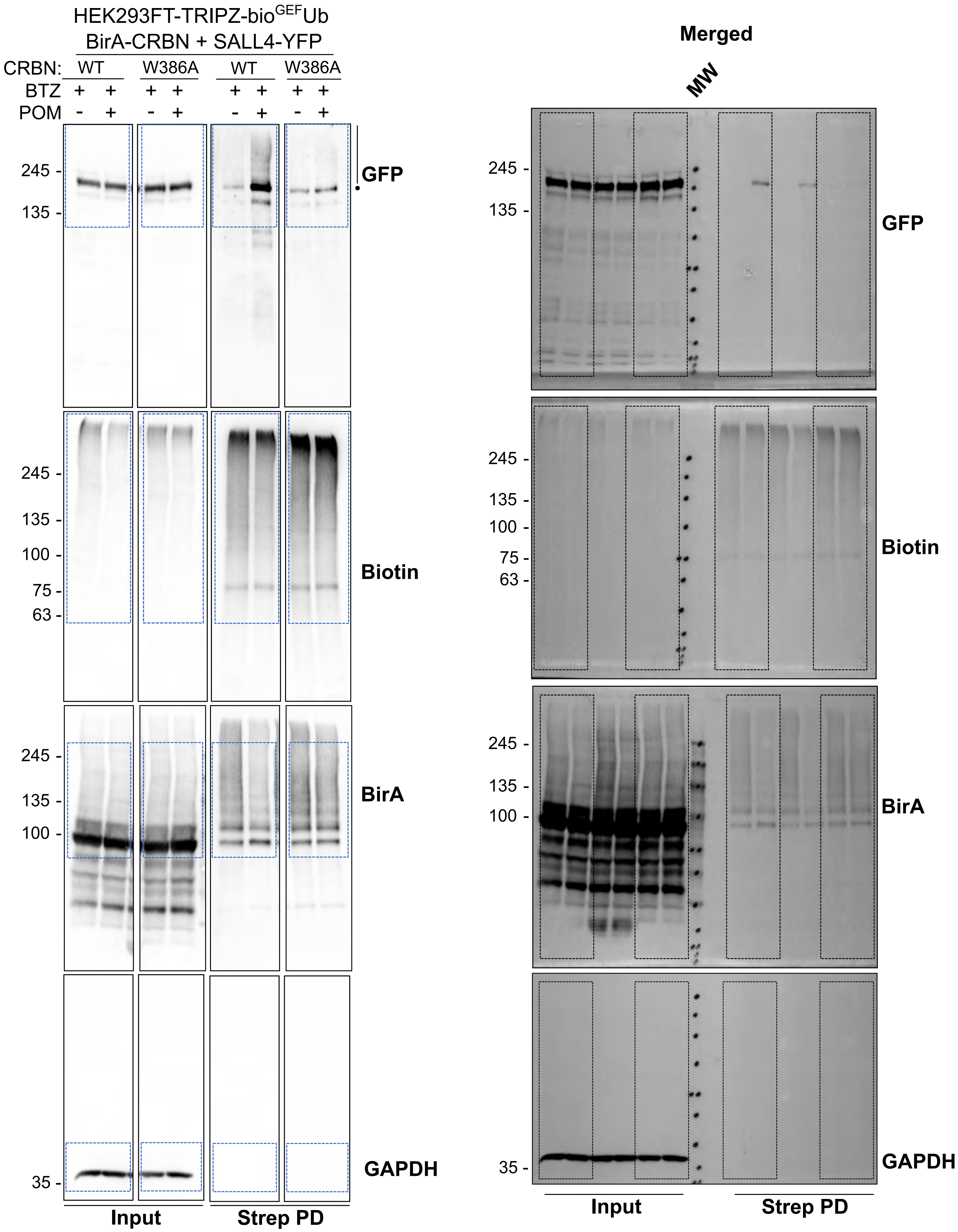

HEK293FT-TRIPZ-bio^{GEF}Ub
BirA-CRBN + SALL4-YFP
CRBN: WT W386A WT W386A
BTZ + + + + + + + +
POM - + - + - + - +
245
135
GFP
245
135
100
75
63
Biotin
245
135
100
BirA
35
GAPDH
Input
Strep PD
Merged
MW
245
135
GFP
245
135
100
75
63
Biotin
245
135
100
BirA
35
GAPDH
Input
Strep PD

Fig. 3a

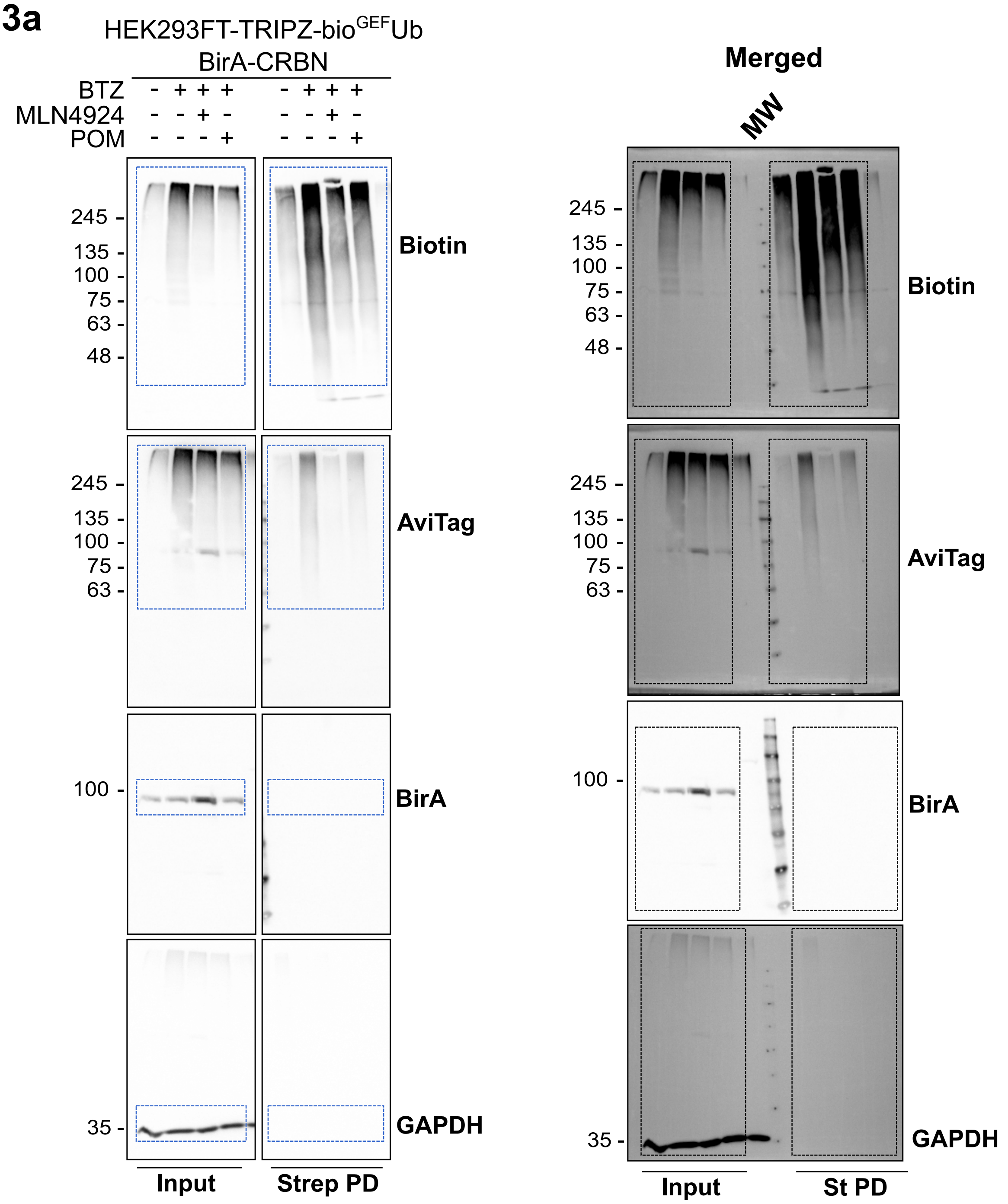
HEK293FT-TRIPZ-bioGEFUb
BirA-CRBN
BTZ - + + + - + + +
MLN4924 - - + - - - + -
POM - - - + - - - +
245
135
100
75
63
48
Biotin
AviTag
BirA
35
GAPDH
Input
Strep PD
Merged
MW
St PD

Fig. 3e

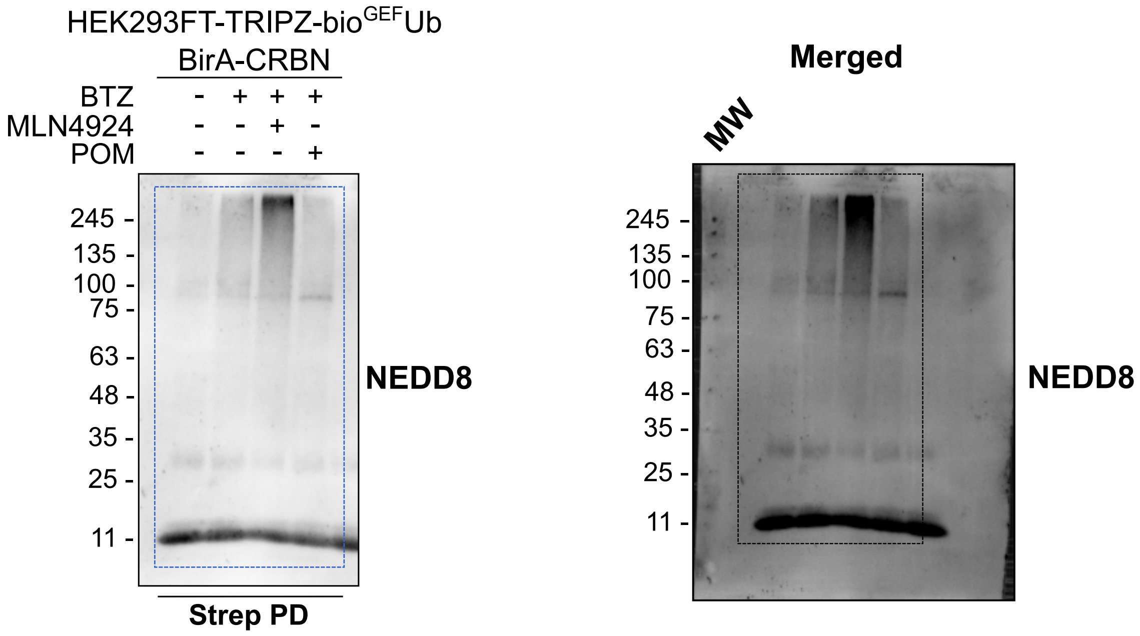
HEK293FT-TRIPZ-bioGEFUb
BirA-CRBN
BTZ - + + +
MLN4924 - - + -
POM - - - +
245
135
100
75
63
48
35
25
11
NEDD8
Strep PD
Merged
MW

Fig. 4b

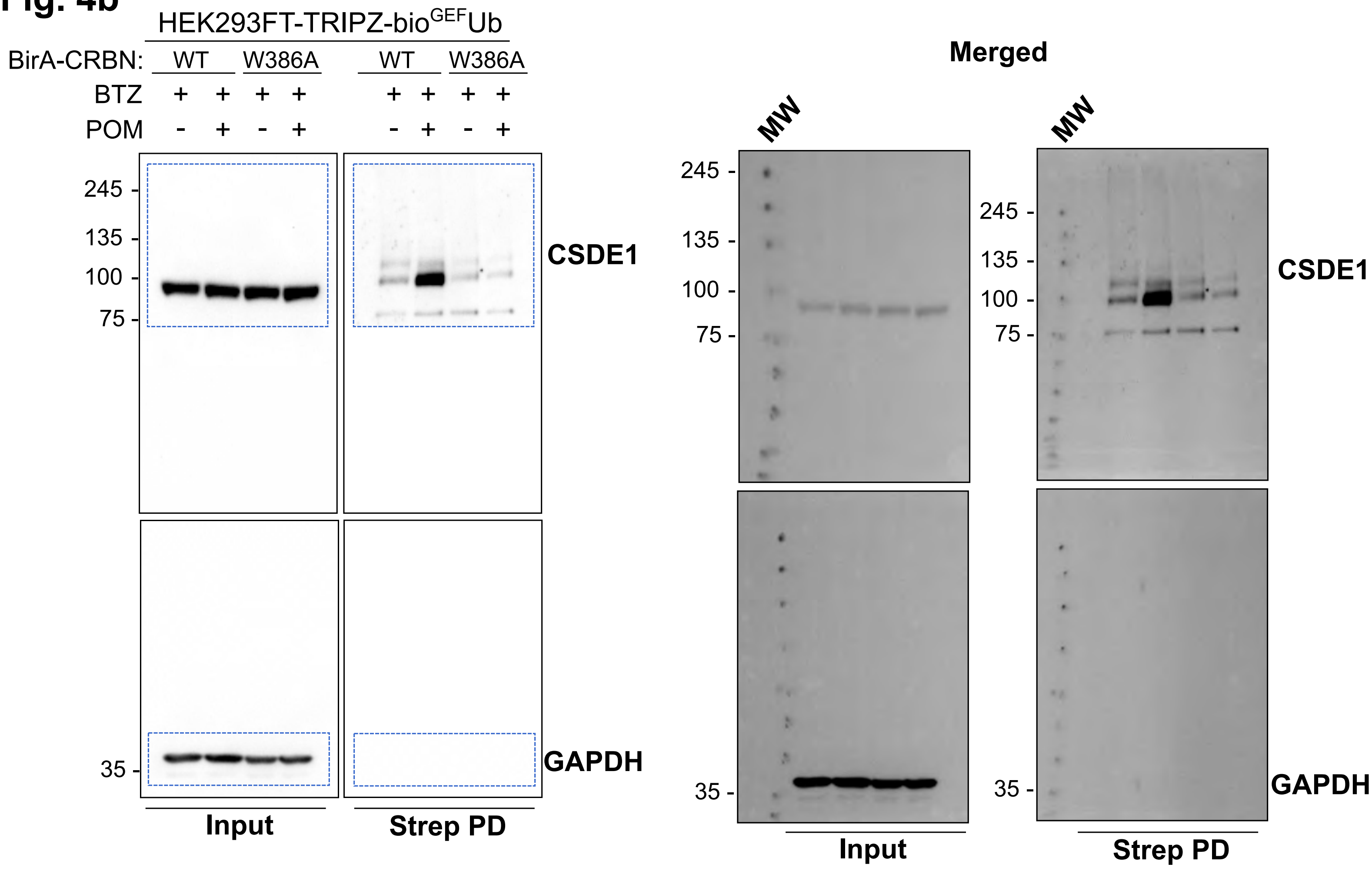
HEK293FT-TRIPZ-bioGEFUb
BirA-CRBN: WT W386A WT W386A
BTZ + + + + + + + +
POM - + - + - + - +
245
135
100
75
35
CSDE1
GAPDH
Input
Strep PD
Merged
MW
MW
245
135
100
75
35
245
135
100
75
35
CSDE1
GAPDH
Input
Strep PD

Fig. 4c

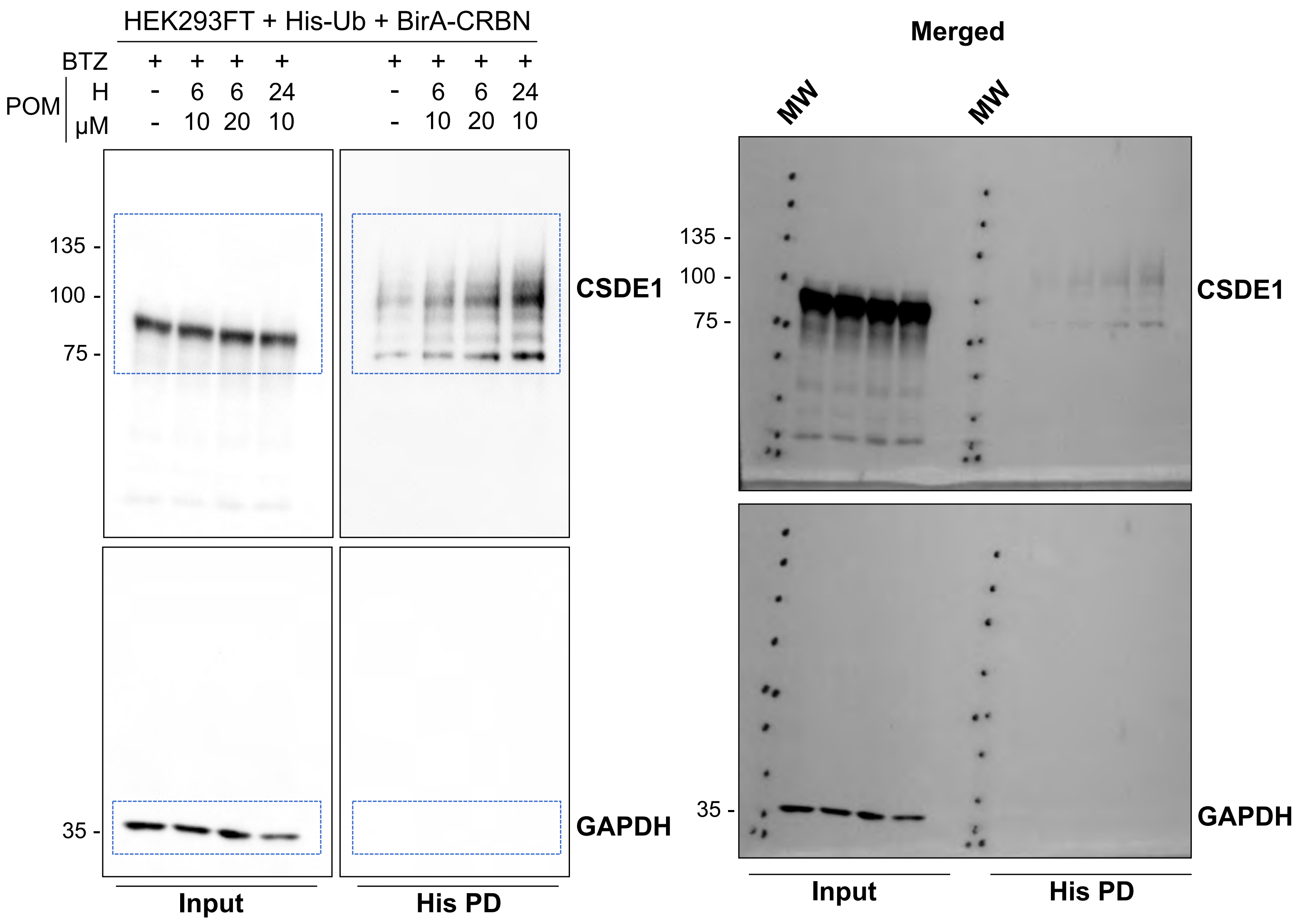
HEK293FT + His-Ub + BirA-CRBN
BTZ + + + + + + + +
POM H - 6 6 24 - 6 6 24
μM - 10 20 10 - 10 20 10
135
100
75
35
CSDE1
GAPDH
Input
His PD
Merged
MW
MW
135
100
75
35
CSDE1
GAPDH
Input
His PD

Fig. 5b

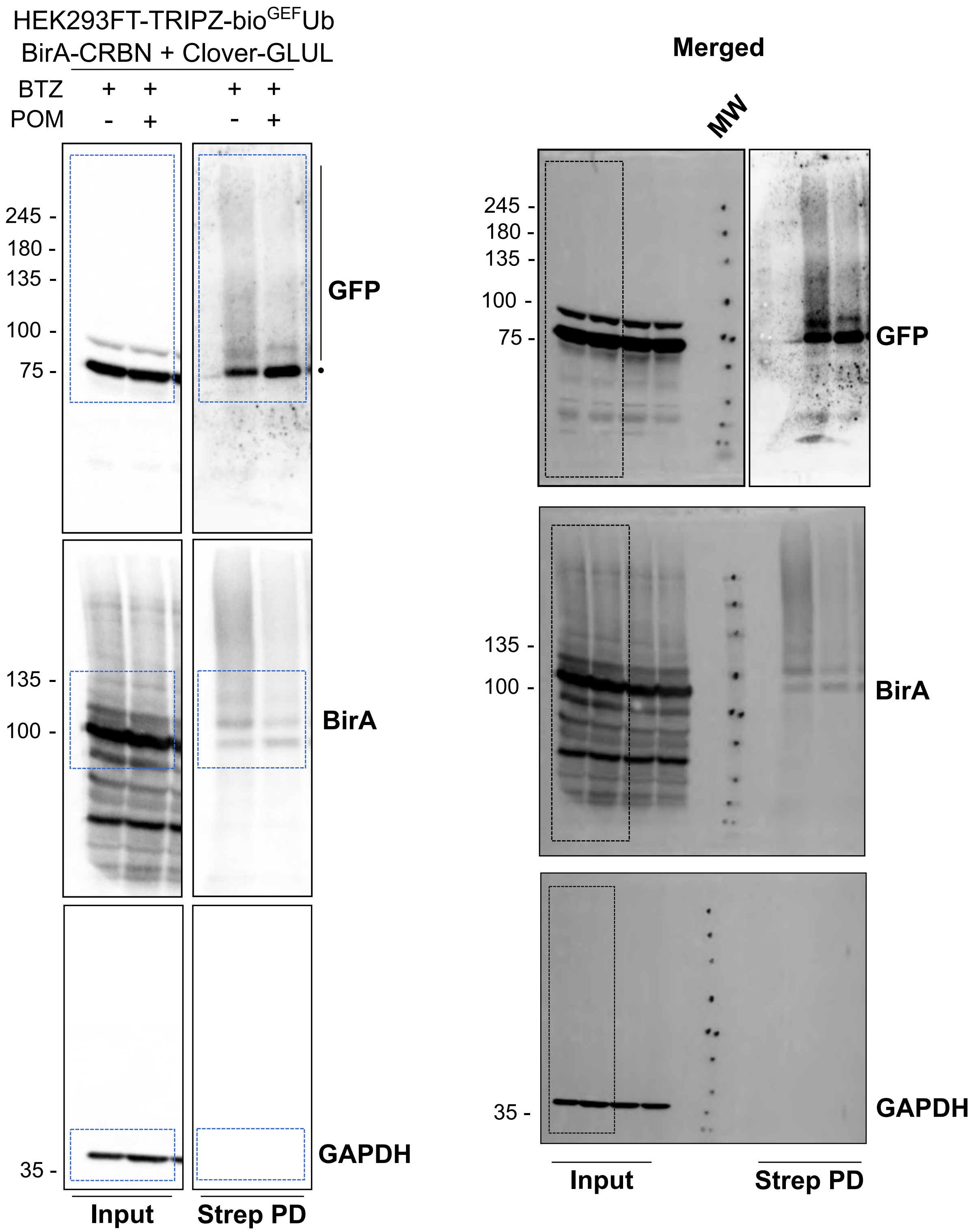

HEK293FT-TRIPZ-bioGEFUb
BirA-CRBN + Clover-GLUL
BTZ + + + +
POM - + - +
245
180
135
100
75
GFP
BirA
35
GAPDH
Input
Strep PD
Merged
MW

Fig. 5c

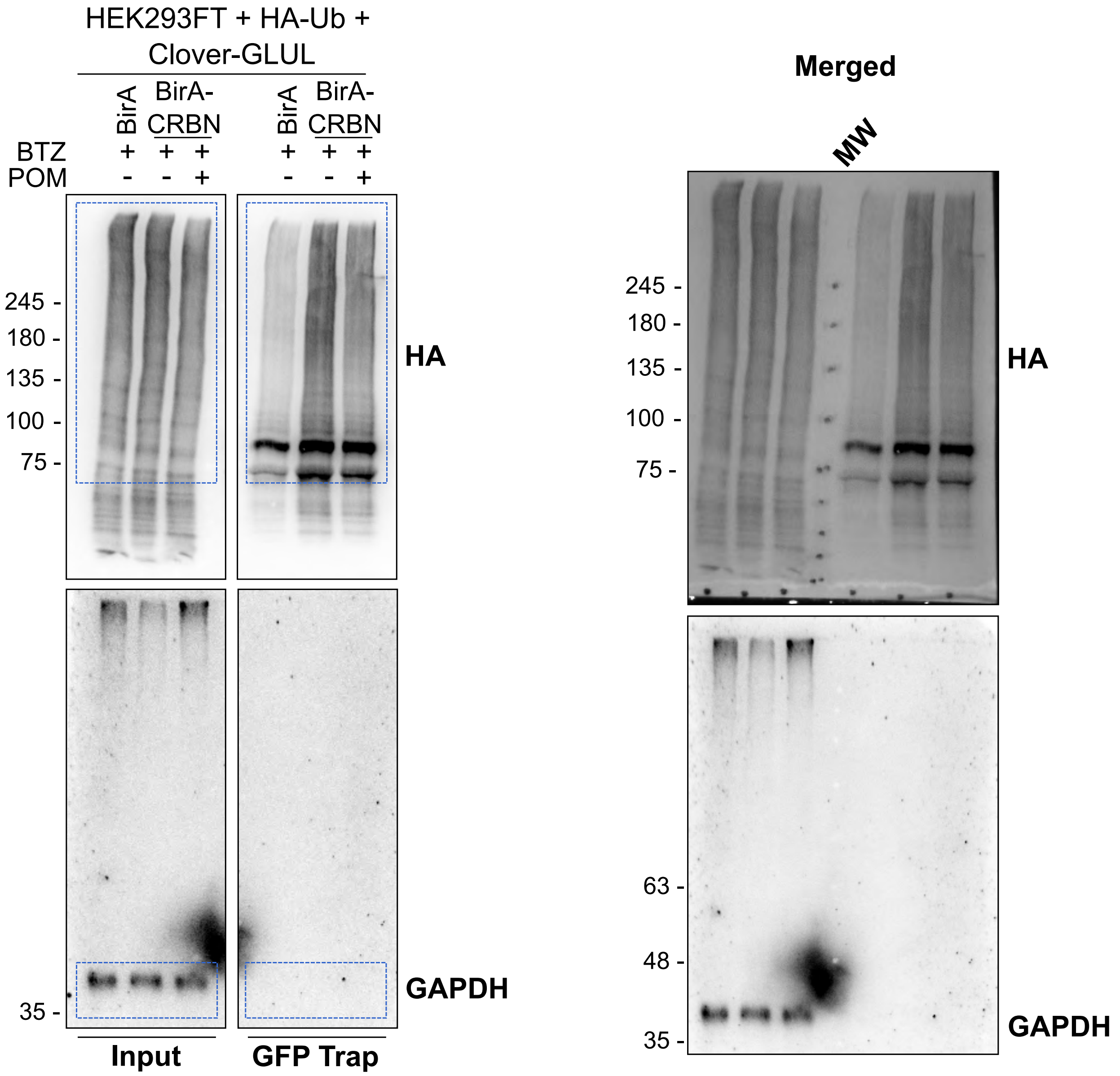
HEK293FT + HA-Ub +
Clover-GLUL
BirA
BirA-
CRBN
BirA
BirA-
CRBN
BTZ + + + + + +
POM - - + - - +
245 -
180 -
135 -
100 -
75 -
HA
35 -
GAPDH
Input
GFP Trap
Merged
MW
245 -
180 -
135 -
100 -
75 -
HA
63 -
48 -
35 -
GAPDH

Supplementary Fig. 1a

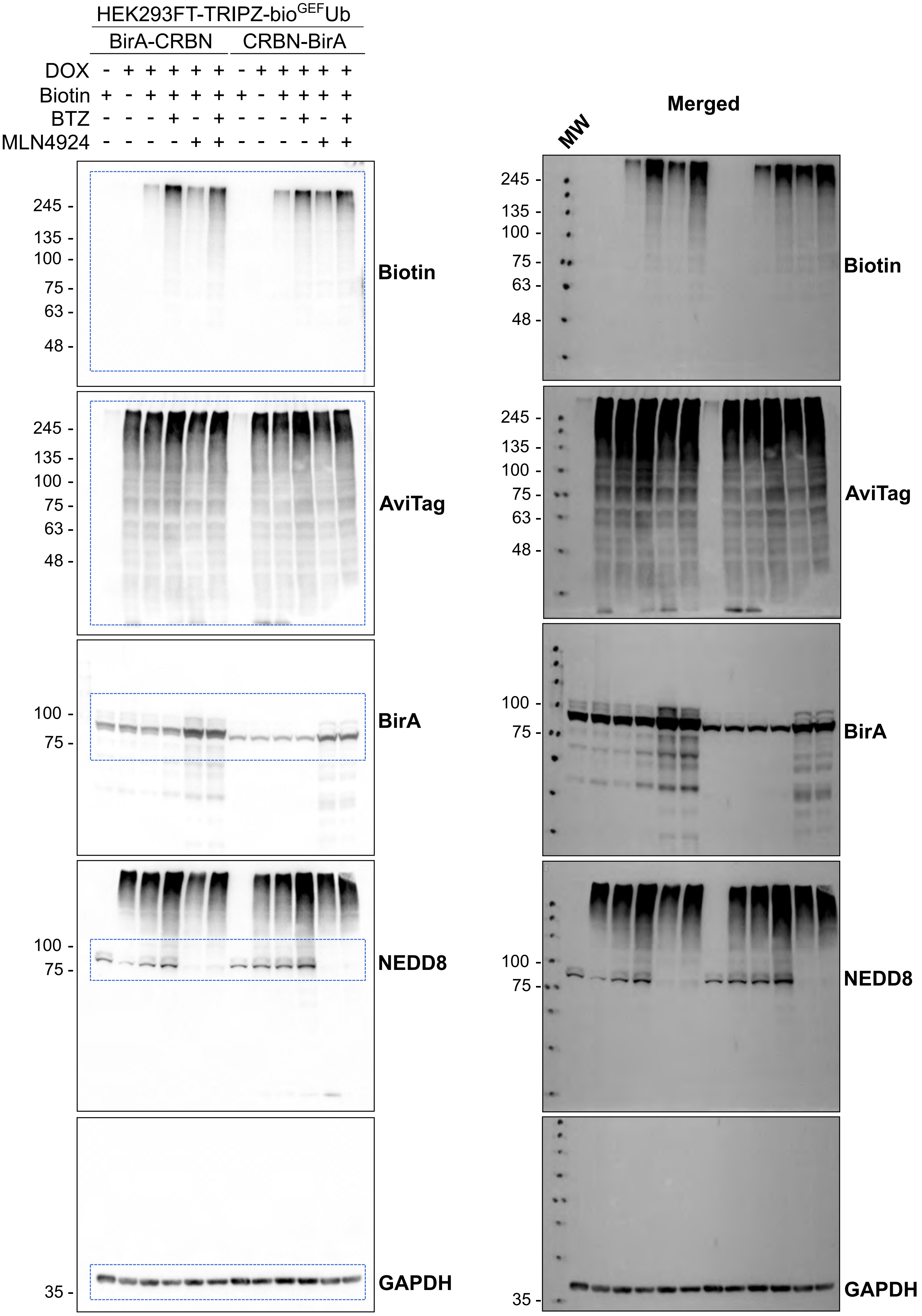

Supplementary Fig. 1b

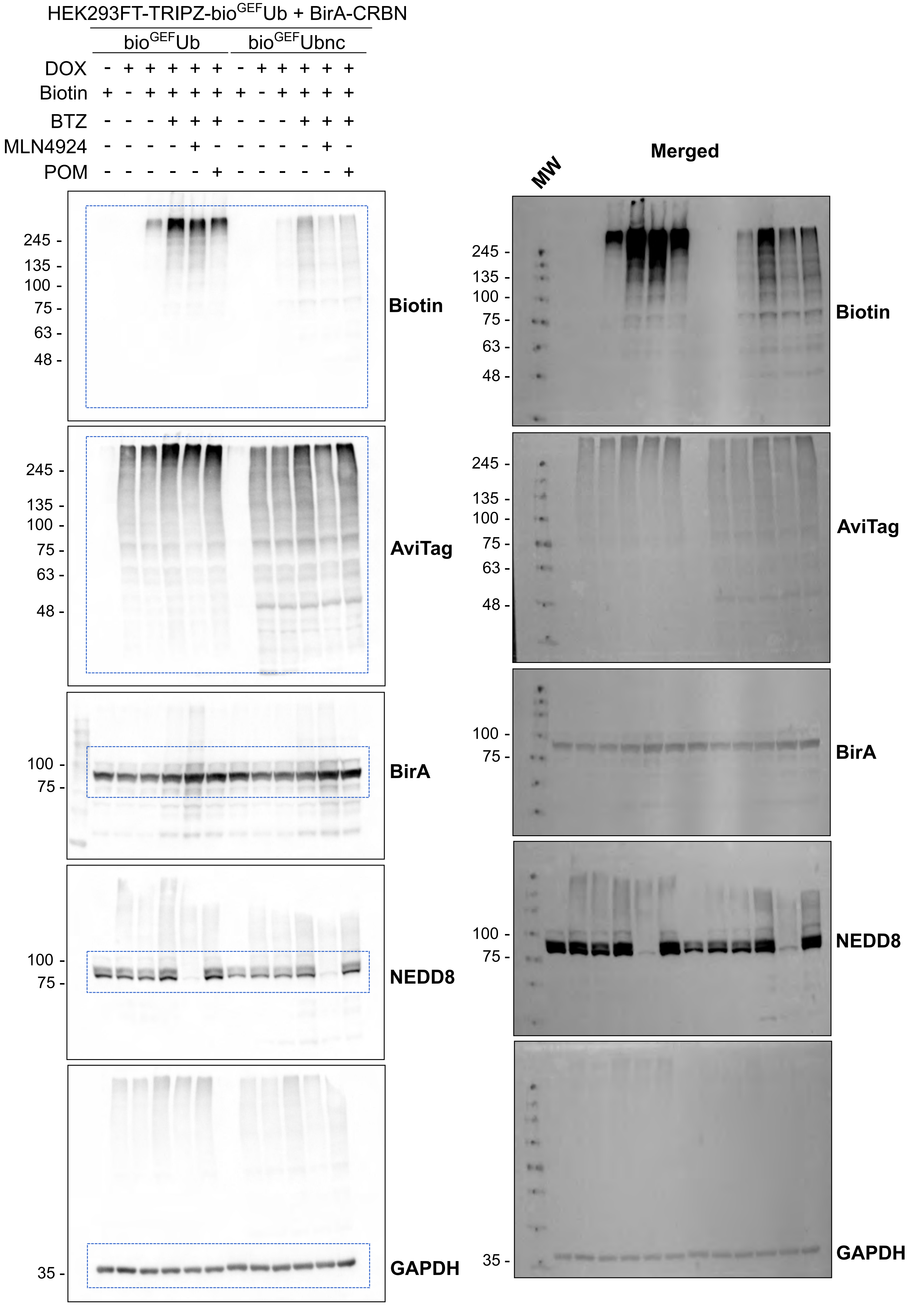

Supplementary Fig. 1c

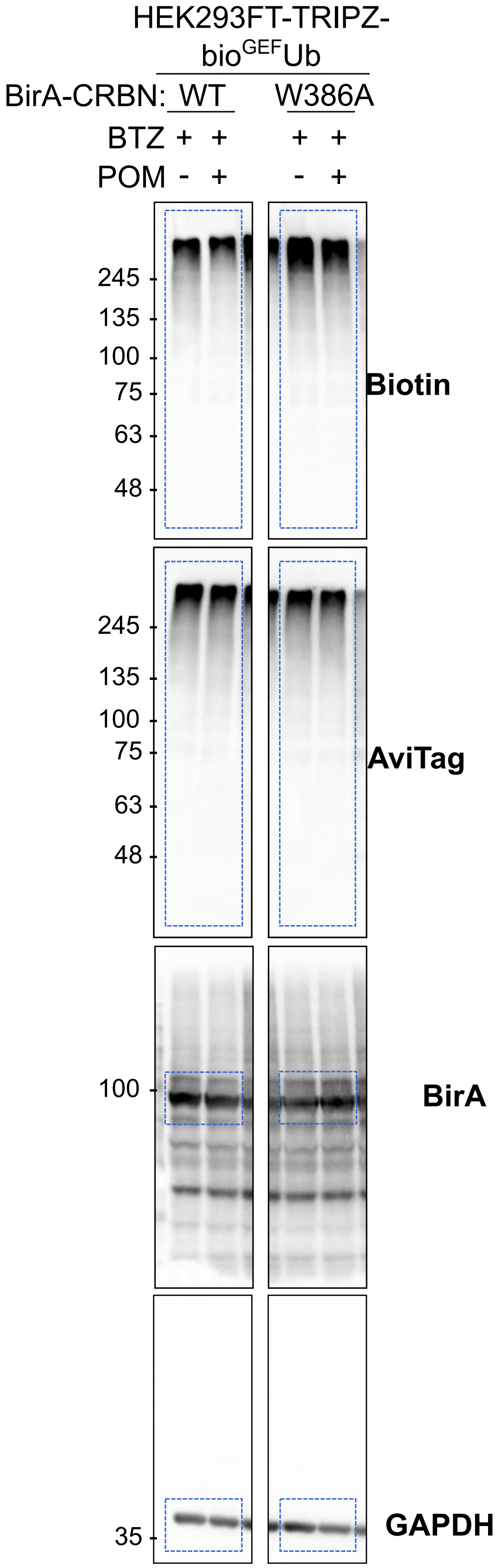

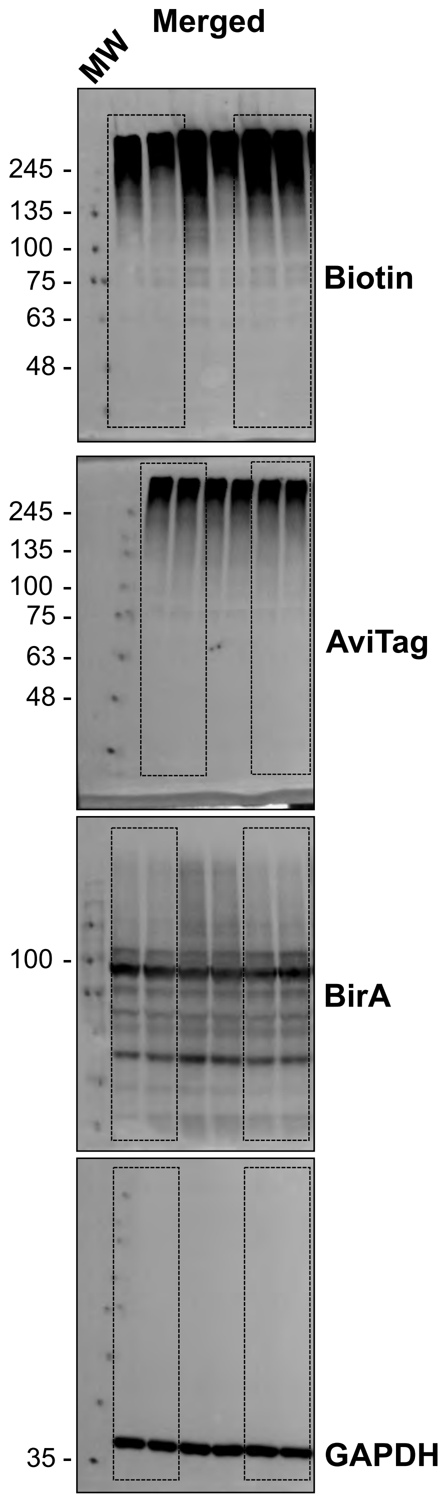

Supplementary Fig. 8a

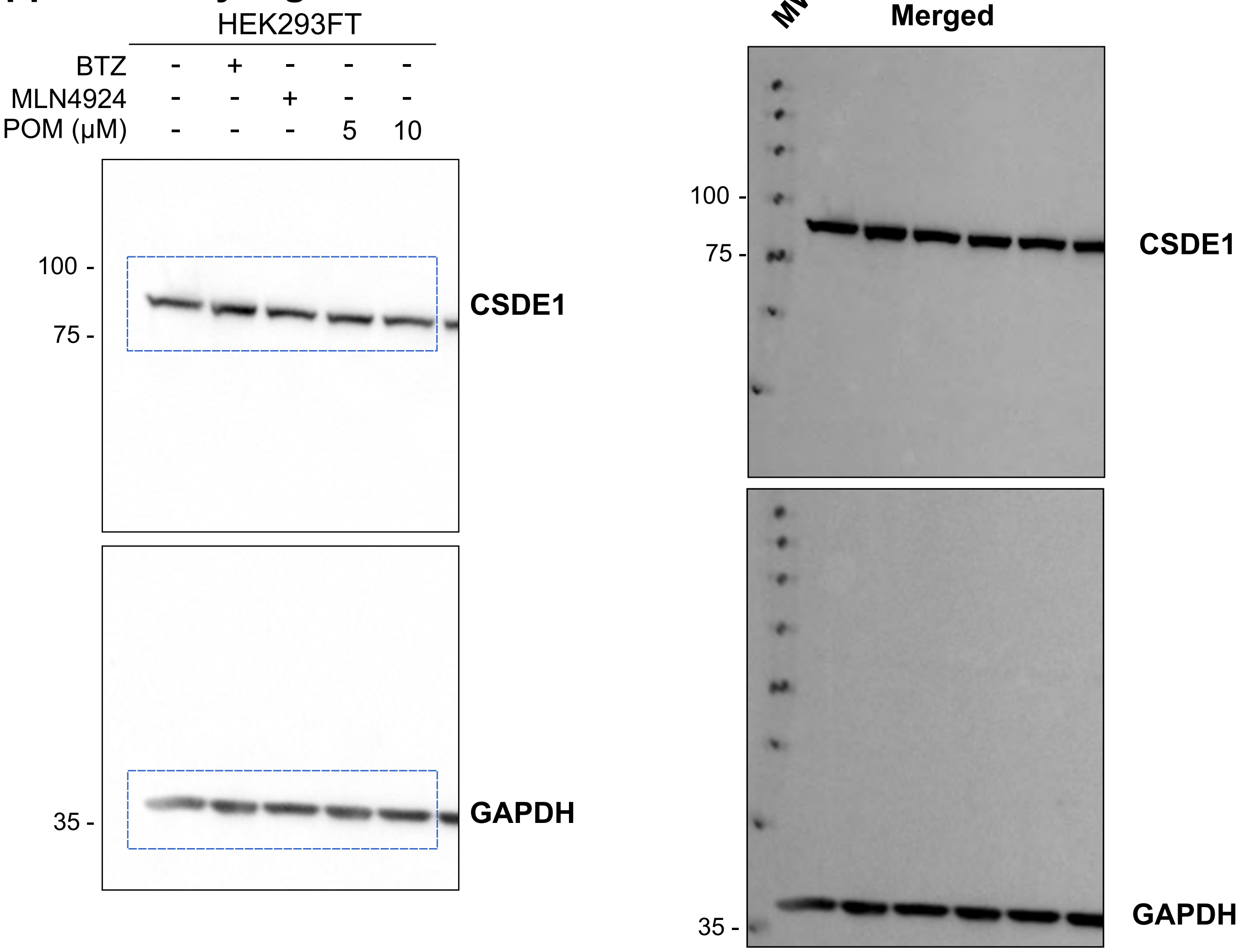

Supplementary Fig. 8c

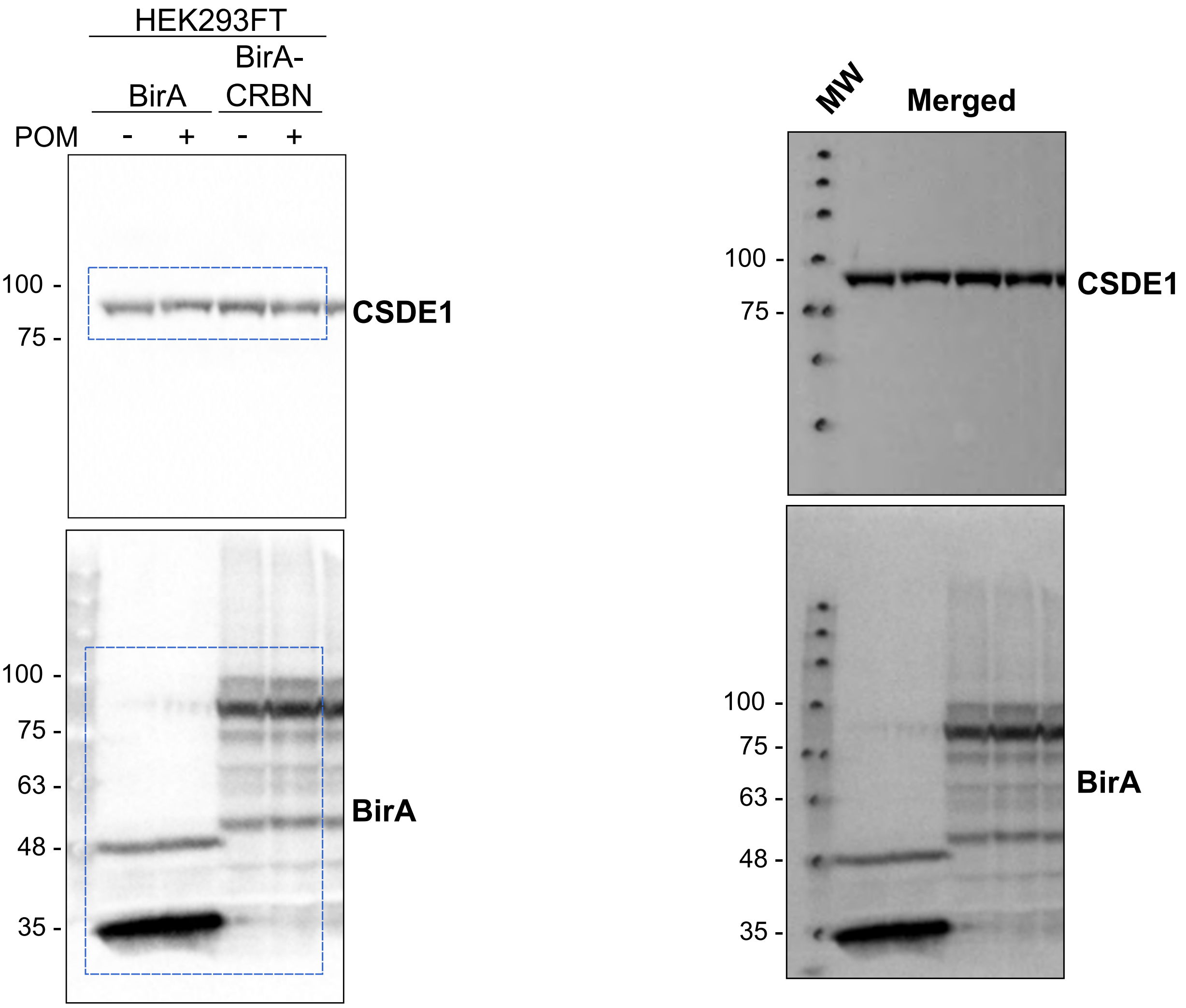

Supplementary Fig. 8e

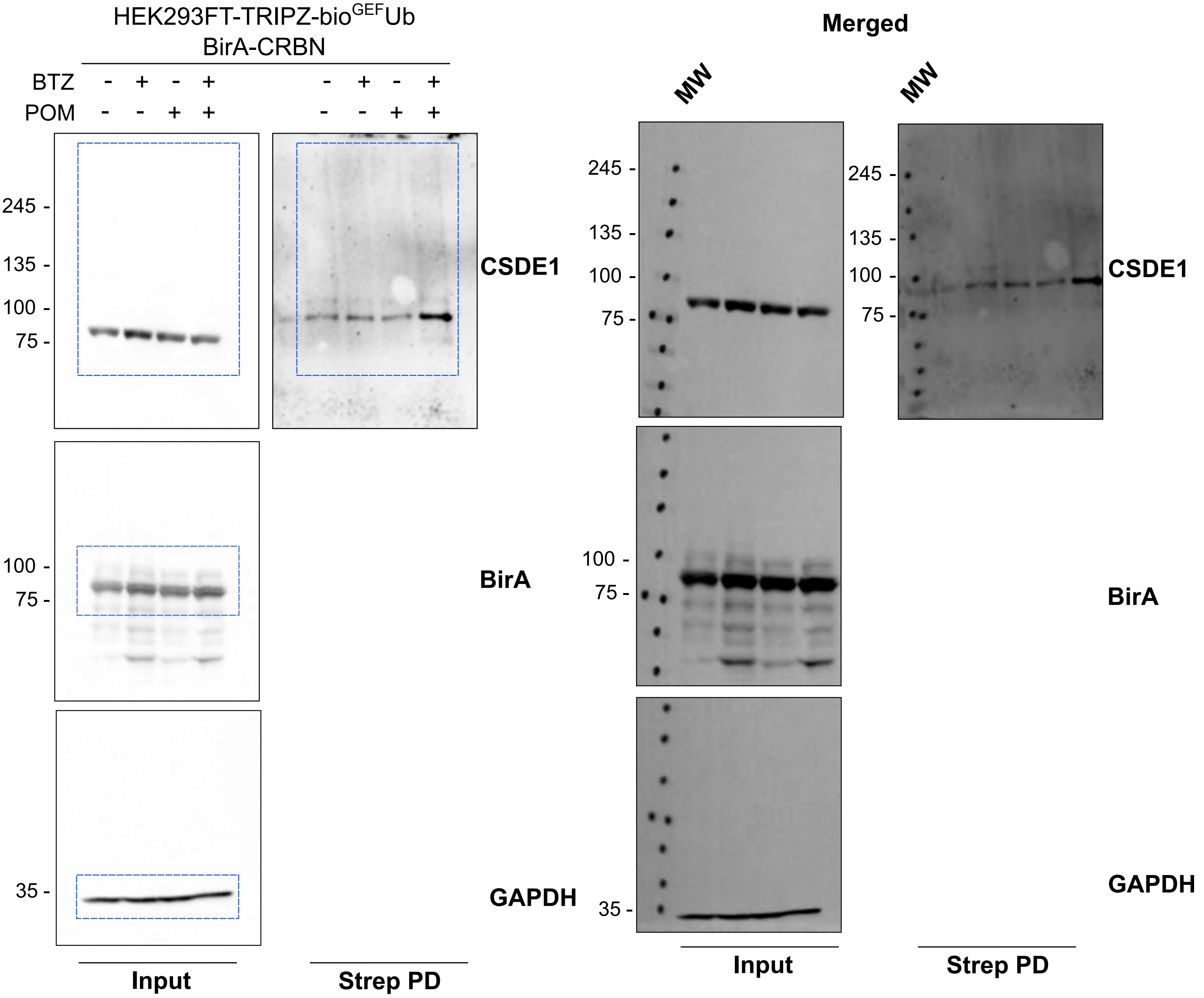

Supplement: Supplementary file 12 — Supplementary Material 12. Uncropped Western blots. Images with the same exposures as used in the main Figures are shown to the left. Merged images show the positioning of the molecular weight markers and might show different exposure times. [file 12964_2025_2091_MOESM12_ESM.pdf]
