# Supplementary material for: Cullin-RING ligase BioE3 reveals molecular-glue-induced neosubstrates and rewiring of the endogenous Cereblon ubiquitome
Source: Cell Commun Signal. 2025 Feb 19;23:101. doi: 10.1186/s12964-025-02091-5 (PMC11841277; doi:10.1186/s12964-025-02091-5)
Supplement: Supplementary file 2 — Supplementary Material 2. Fig. S2. STRING network analysis of CRBN ubiquitinated substrates. Substrates defined in Figure 3b show a highly interconnected network composed of 71% of the proteins. Highly interconnected sub-clusters were derived and characterized using MCODE. Color, transparency and size of the nodes were discretely mapped to the Log2 enrichment value as indicated. [file 12964_2025_2091_MOESM2_ESM.pdf]

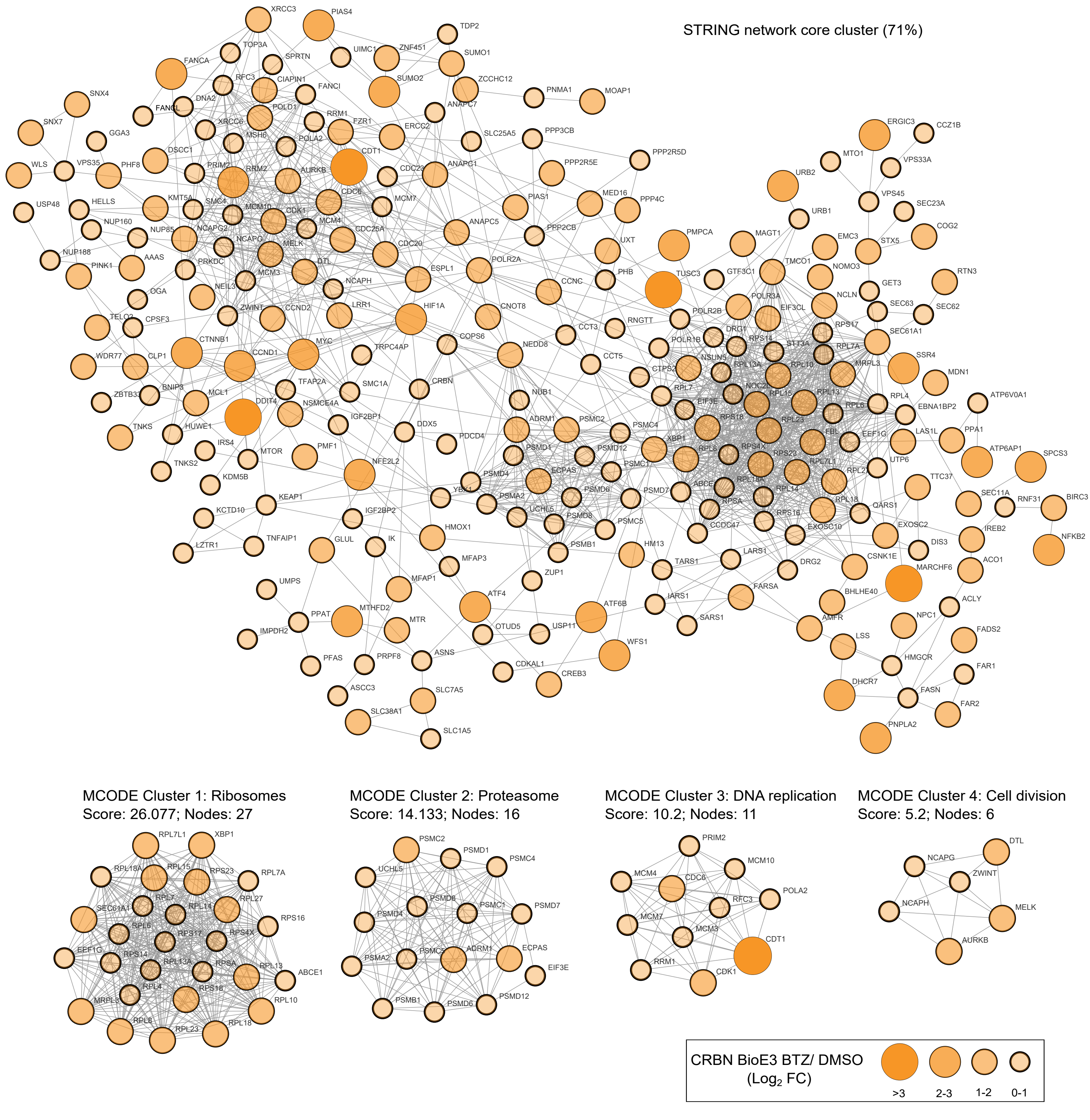
STRING network core cluster (71%)
XRCC3
PIAS4
TDP2
TOP3A
UIMC1
ZNF451
SUMO1
SPRTN
FANCA
RFC3
CIAPIN1
SUMO2
ZCCHC12
FANCI
PNMA1
MOAP1
SNX4
DNA2
FANCL
POLD1
ANAPC7
SNX7
XRCC6
RRM1
FZR1
ERCC2
ERGIC3
CCZ1B
GGA3
MSH6
POLA2
SLC25A5
PPP3CB
MTO1
WLS
VPS35
PHF8
DSCC1
PRIM2
RRM2
CDT1
CDC23
ANAPC1
PPP2R5E
PPP2R5D
VPS33A
URB2
USP48
HELLS
KMT5A
SMC4
AURKB
CDC6
MCM7
PIAS1
MED16
PPP4C
VPS45
SEC23A
NUP160
MCM10
CDK1
MCM4
URB1
NUP85
NCAPG2
NCAPG
MELK
CDC25A
ANAPC5
PPP2CB
COG2
NUP188
CDC20
UXT
PMPCA
MAGT1
EMC3
STX5
PINK1
AAAS
PRKDC
DTL
MCM3
NCAPH
ESPL1
POLR2A
PHB
TUSC3
GTF3C1
TMCO1
NOMO3
RTN3
OGA
NEIL3
CCNC
GET3
ZWINT
CCND2
LRR1
HIF1A
CNOT8
POLR2B
POLR3A
EIF3CL
NCLN
SEC63
SEC62
TELO2
CPSF3
CCT3
RNGTT
DRG1
RPS17
CTNNB1
MYC
COPS6
POLR1B
RPS14
STT3A
RPL7A
SEC61A1
WDR77
CLP1
CCND1
TRPC4AP
NEDD8
CCT5
NSUN5
RPL13A
RPL10
MRPL3
SSR4
MDN1
TFAP2A
CRBN
CTPS2
NOC2L
RPL15
RPL13
ZBTB33
BNIP3
MCL1
SMC1A
NUB1
RPL7
RPL4
EBNA1BP2
ATP6V0A1
DDIT4
NSMCE4A
EIF3E
RPL6
IGF2BP1
ADRM1
PSMC2
RPS18
RPL23
TNKS
HUWE1
DDX5
PSMC4
XBP1
FBL
EEF1G
LAS1L
PPA1
PDCD4
PSMD1
PSMD12
RPL8
RPS4X
IRS4
MTOR
PMF1
ATP6AP1
SPCS3
TNKS2
NFE2L2
PSMD4
ECPAS
PSMC1
RPS23
RPL7L1
RPL27
KDM5B
YBX1
PSMD6
PSMD7
ABCE1
RPL18A
RPL14
UTP6
TTC37
KEAP1
PSMA2
UCHL5
RPSA
RPL18
QARS1
SEC11A
RNF31
BIRC3
KCTD10
IGF2BP2
PSMD8
PSMC5
CCDC47
RPS16
EXOSC10
EXOSC2
IREB2
TNFAIP1
GLUL
IK
HMOX1
PSMB1
DIS3
NFKB2
LZTR1
HM13
TARS1
LARS1
DRG2
CSNK1E
MFAP3
ACO1
MARCHF6
ZUP1
UMPS
MFAP1
ATF4
FARSA
PPAT
MTHFD2
ATF6B
IARS1
BHLHE40
ACLY
MTR
OTUD5
SARS1
AMFR
NPC1
IMPDH2
USP11
FADS2
LSS
WFS1
ASNS
PFAS
PRPF8
HMGCR
CDKAL1
FAR1
CREB3
DHCR7
ASCC3
SLC7A5
FASN
SLC38A1
FAR2
SLC1A5
PNPLA2
MCODE Cluster 1: Ribosomes
Score: 26.077; Nodes: 27
RPL7L1
XBP1
RPL18A
RPL15
RPS23
RPL7A
RPL7
RPL14
RPL27
SEC61A1
RPS16
RPL6
RPS17
RPS4X
EEF1G
RPS14
RPL13A
RPSA
RPL13
ABCE1
RPL4
RPS18
MRPL3
RPL10
RPL8
RPL23
RPL18
MCODE Cluster 2: Proteasome
Score: 14.133; Nodes: 16
PSMC2
PSMD1
PSMC4
UCHL5
PSMD8
PSMC1
PSMD7
PSMD4
PSMA2
PSMC5
ADRM1
ECPAS
EIF3E
PSMB1
PSMD6
PSMD12
MCODE Cluster 3: DNA replication
Score: 10.2; Nodes: 11
PRIM2
MCM10
MCM4
CDC6
POLA2
RFC3
MCM7
MCM3
CDT1
RRM1
CDK1
MCODE Cluster 4: Cell division
Score: 5.2; Nodes: 6
DTL
NCAPG
ZWINT
NCAPH
MELK
AURKB
CRBN BioE3 BTZ/ DMSO
(Log2 FC)
>3
2-3
1-2
0-1
