# Supplementary material for: Cullin-RING ligase BioE3 reveals molecular-glue-induced neosubstrates and rewiring of the endogenous Cereblon ubiquitome
Source: Cell Commun Signal. 2025 Feb 19;23:101. doi: 10.1186/s12964-025-02091-5 (PMC11841277; doi:10.1186/s12964-025-02091-5)
Supplement: Supplementary file 6 — Supplementary Material 6. Fig. S6. STRING network analysis of CRBN neosubstrates. The neosubstrates upon pomalidomide treatment defined in Figure 4a show a highly interconnected network composed of 71% of the proteins. Highly interconnected sub-clusters were derived and characterized using MCODE. Color, transparency and size of the nodes were discretely mapped to the Log2 enrichment value as indicated. [file 12964_2025_2091_MOESM6_ESM.pdf]

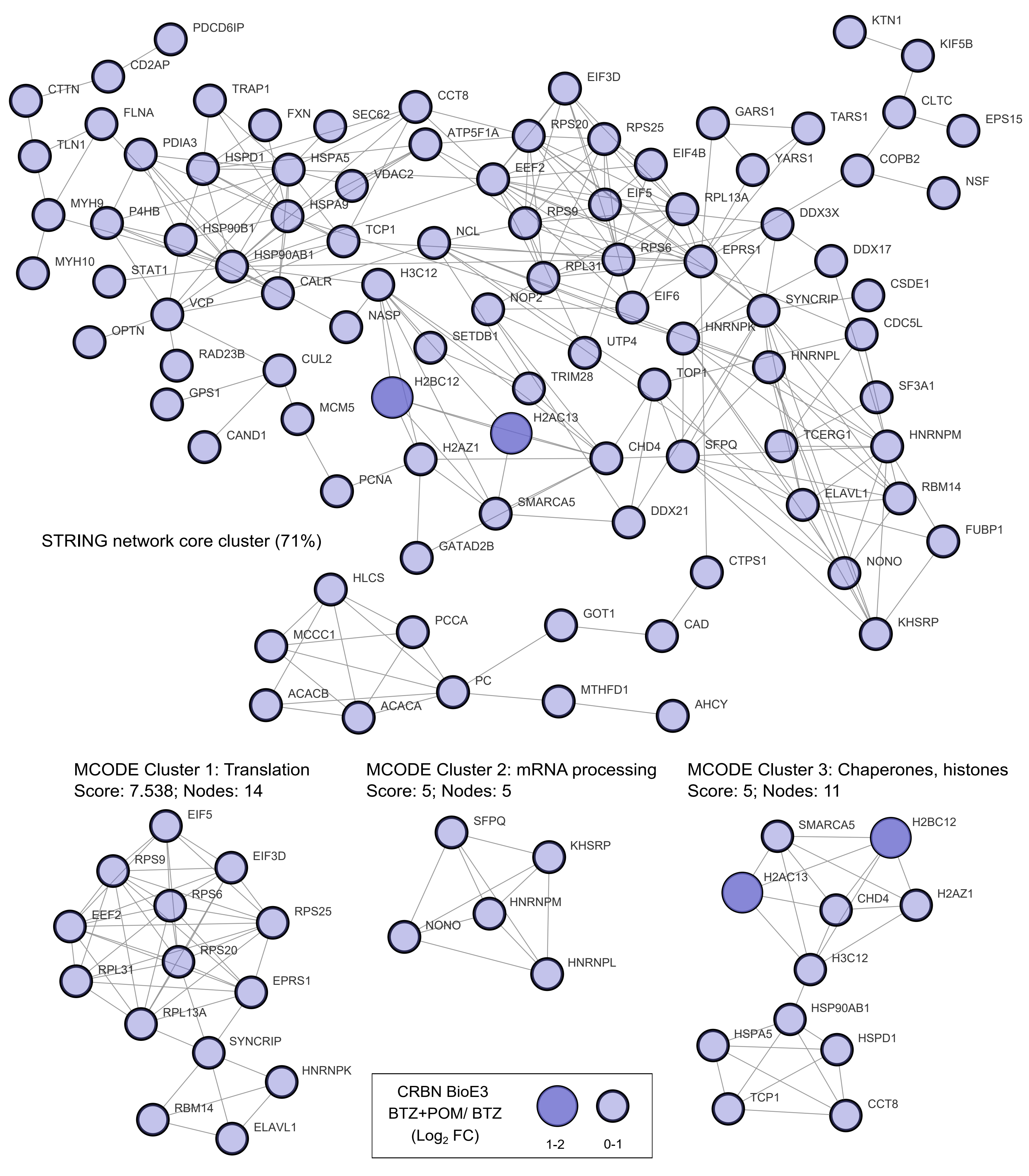
STRING network core cluster (71%)
PDCD6IP
CD2AP
CTTN
TLN1
FLNA
MYH9
MYH10
TRAP1
FXN
SEC62
CCT8
ATP5F1A
PDIA3
HSPD1
HSPA5
VDAC2
P4HB
HSP90B1
HSPA9
TCP1
STAT1
HSP90AB1
CALR
VCP
OPTN
RAD23B
GPS1
CUL2
CAND1
MCM5
PCNA
NCL
H3C12
NASP
SETDB1
H2BC12
H2AC13
H2AZ1
SMARCA5
GATAD2B
TRIM28
NOP2
UTP4
EIF3D
RPS20
RPS25
EIF4B
EEF2
EIF5
RPS9
RPL13A
RPS6
RPL31
EIF6
EPRS1
TOP1
CHD4
DDX21
SFPQ
HNRNPK
HNRNPL
SYNCRIP
GARS1
TARS1
YARS1
KTN1
KIF5B
CLTC
EPS15
COPB2
NSF
DDX3X
DDX17
CSDE1
CDC5L
SF3A1
TCERG1
HNRNPM
ELAVL1
RBM14
FUBP1
NONO
KHSRP
CTPS1
CAD
GOT1
HLCS
PCCA
MCCC1
PC
ACACB
ACACA
MTHFD1
AHCY
MCODE Cluster 1: Translation
Score: 7.538; Nodes: 14
EIF5
RPS9
EIF3D
RPS6
EEF2
RPS25
RPS20
RPL31
EPRS1
RPL13A
SYNCRIP
HNRNPK
RBM14
ELAVL1
MCODE Cluster 2: mRNA processing
Score: 5; Nodes: 5
SFPQ
KHSRP
HNRNPM
NONO
HNRNPL
MCODE Cluster 3: Chaperones, histones
Score: 5; Nodes: 11
SMARCA5
H2BC12
H2AC13
CHD4
H2AZ1
H3C12
HSP90AB1
HSPA5
HSPD1
TCP1
CCT8
CRBN BioE3
BTZ+POM/ BTZ
(Log2 FC)
1-2
0-1
